# Patients with type 1 Gaucher disease in South Florida, USA: demographics, genotypes, disease severity and treatment outcomes

**DOI:** 10.1186/1750-1172-9-45

**Published:** 2014-03-31

**Authors:** Marissa Orenstein, Deborah Barbouth, Olaf A Bodamer, Neal J Weinreb

**Affiliations:** 1 Department of Medical Education, Miller School of Medicine, Miami, FL, USA; 2 Dr John T. Macdonald Foundation Department of Human Genetics, Miller School of Medicine, Miami, FL, USA; 3 Hussman Institute of Human Genomics University of Miami, Miller School of Medicine, Miami, FL, USA; 4 University Research Foundation for Lysosomal Storage Diseases, Inc., Northwest Oncology Hematology Associates PA, 8170 Royal Palm Boulevard, Coral Springs, FL 33065, USA

**Keywords:** Gaucher disease, Glucocerebrosidase, Phenotype, Genotype, South Florida, Natural history, Enzyme replacement therapy, Treatment outcomes, Severity score

## Abstract

**Background:**

Gaucher disease, an autosomal recessive condition due to deficiency of lysosomal glucocerebrosidase, is a multisystemic disease, with variable age of onset, severity and progression. It is classified into subtypes delineated by the absence (type 1) or presence (type 2 and 3) of primary nervous system involvement.

The ethnically diverse, largely immigrant population in South Florida has a spectrum of Gaucher disease phenotypes, creating a challenge for optimization of disease management and an opportunity to explore treatment patterns.

**Methods:**

Ninety-three records from patients with Gaucher type I in South Florida were retrieved from the International Collaborative Gaucher Group (ICGG) Registry. Individual genotypes were correlated with severity scores and success at achieving published therapeutic goals for haemoglobin concentration, platelet count, spleen volume, liver volume and amelioration of bone pain and bone crises.

**Results:**

The majority of patients were diagnosed during the fifth decade of life. Almost two-thirds were homozygous for the N370S mutation, reflecting the large Ashkenazi Jewish population in South Florida. The majority received imiglucerase (62.8%) at various intervals. 24.5% of patients underwent splenectomy before starting enzyme replacement therapy. After a median 12 treatment years, South Florida patients matched or exceeded the ICCG 4 year therapeutic goal achievement for platelet count (85.4% vs. 79.6% success), spleen volume (93.3% vs. 78.0% success), liver volume (93.4% vs. 90.6% success), and bone crises (100% vs. 99% success). Nevertheless, fewer patients with intact spleens had sustained achievement of all 6 therapeutic goals (30.4% versus 41.4%) and only 40% of the splenectomy patients sustained achievement of 5/5 possible goals. 54.7% of the intact spleen patients continued to have bone pain vs. 29.8% in ICCG. Significantly, only 37% of the ICGG patient cohort had bone pain prior to initiation of treatment compared to 73.4% of the South Florida patients (moderate or severe pain in 59.6%).

**Conclusions:**

Demographic characteristics are a significant determinant of the differences in response to treatment observed in South Florida Gaucher patients compared to those described in the international population enrolled in the ICGG Gaucher Registry. Individual genotypes and ethnic background are important considerations for optimizing patient care for Gaucher disease.

## Introduction

Gaucher disease (GD) is an autosomal recessive lysosomal storage disorder that, with the exception of rare patients with saposin C deficiency, is caused by mutations in the glucocerebrosidase gene, *GBA1*[1]. Deficient glucocerebrosidase activity leads to accumulation of the enzyme’s substrate, glucocerebroside (glucosylceramide), in tissue macrophages primarily in the liver, spleen and bone marrow. Subject to genotype, other genetic modifiers [2,3] and undefined genetic, epigenetic and environmental factors, untreated patients with GD may be asymptomatic with few signs of disease or present with combinations of hematologic abnormalities, hepatosplenomegaly, skeletal disease, growth retardation, and decreased health-related quality of life [1]. GD type 1 (GD1), found in approximately 90% of known GD patients worldwide, is differentiated from GD type 2 and GD type 3 by the absence of overt, early onset neurological signs and symptoms. However, distinct late onset neurological symptoms such as peripheral neuropathy and Parkinson disease may occur in GD1 [4].

The incidence of GD1is estimated at 1/50,000-75,000 live births in non-Jewish populations in North America, Europe and Australia [5,6]. Of more than 350 *GBA1* variant alleles that are associated with GD, 6 mutations account for 98% of those found in the Ashkenazi Jewish population in which GD1 is especially prevalent [7]. One in 10–15 Ashkenazi Jews is a carrier for a *GBA1* mutation projecting a disease incidence of 1/600 live births [8]. However, the observed disease prevalence among North American Ashkenazi Jews is substantially lower than predicted. In South Florida, home to an estimated 500,000 Ashkenazi Jews [9], only approximately 100 Ashkenazi Jews with GD1 have been identified over a 20 year period (NJW, personal observation). This lower than expected prevalence is comparable to other US metropolitan areas with large Ashkenazi Jewish populations [10,11]. The difference between observed and predicted cases with GD1 may be readily explained by the highly variable penetrance resulting in large numbers of asymptomatic individuals who never come to medical attention although missed diagnoses cannot be excluded [12]. Among western non-Jewish populations, the prevalence of GD1 is also less than predicted: 1/150,000-300,000 in French and Spanish national registries [13,14]. It is unclear to what extent phenotypic heterogeneity contributes to this finding in light of evidence that the GD1 clinical phenotype tends to be more severe in non-Jewish patients compared to Ashkenazi Jews [15].

Here, we present a demographic and genotypic profile for 93 GD1 patients who live in South Florida and are enrolled in the ICGG Gaucher Registry and correlate genotype with validated severity scores and hematologic, visceral and skeletal therapeutic outcomes.

## Methods

### Patient population

The ICGG Gaucher Registry was launched in 1991 to track the clinical, demographic, genetic, biochemical and therapeutic characteristics of patients with GD throughout the world, irrespective of disease severity, treatment status or treatment choice [16]. Governance and scientific direction is provided by an international group of physician experts in GD, with operational support from Genzyme, a Sanofi company (Cambridge, Massachusetts). For this report, we independently analyzed the medical records of all patients with Type 1 GD at the University Research Foundation for Lysosomal Storage Diseases South Florida site who enrolled patients into the ICGG Gaucher Registry from 1991-June 2011. All participating patients gave informed consent to participate in the ICCG Registry using forms approved at the time of their enrollment by the Western Institutional Review Board.

### Genotype

Genotype was obtained for most patients by PCR specific oligonucleotide screening for 5 common mutations prevalent in the Ashkenazi Jewish population (N370S, L444P, 84GG, IVS2 + 1, R496H; Genzyme Genetics, acquired by Laboratory Corporation of America in May 2010). Samples from some patients with unidentified alleles were referred to the ICGG Registry Genotyping Service at the laboratory of H. Ronald Scott, MD, PhD at the University of Washington, Seattle, WA, USA for whole gene sequencing.

### Gaucher disease severity scoring

**Figure 1:**
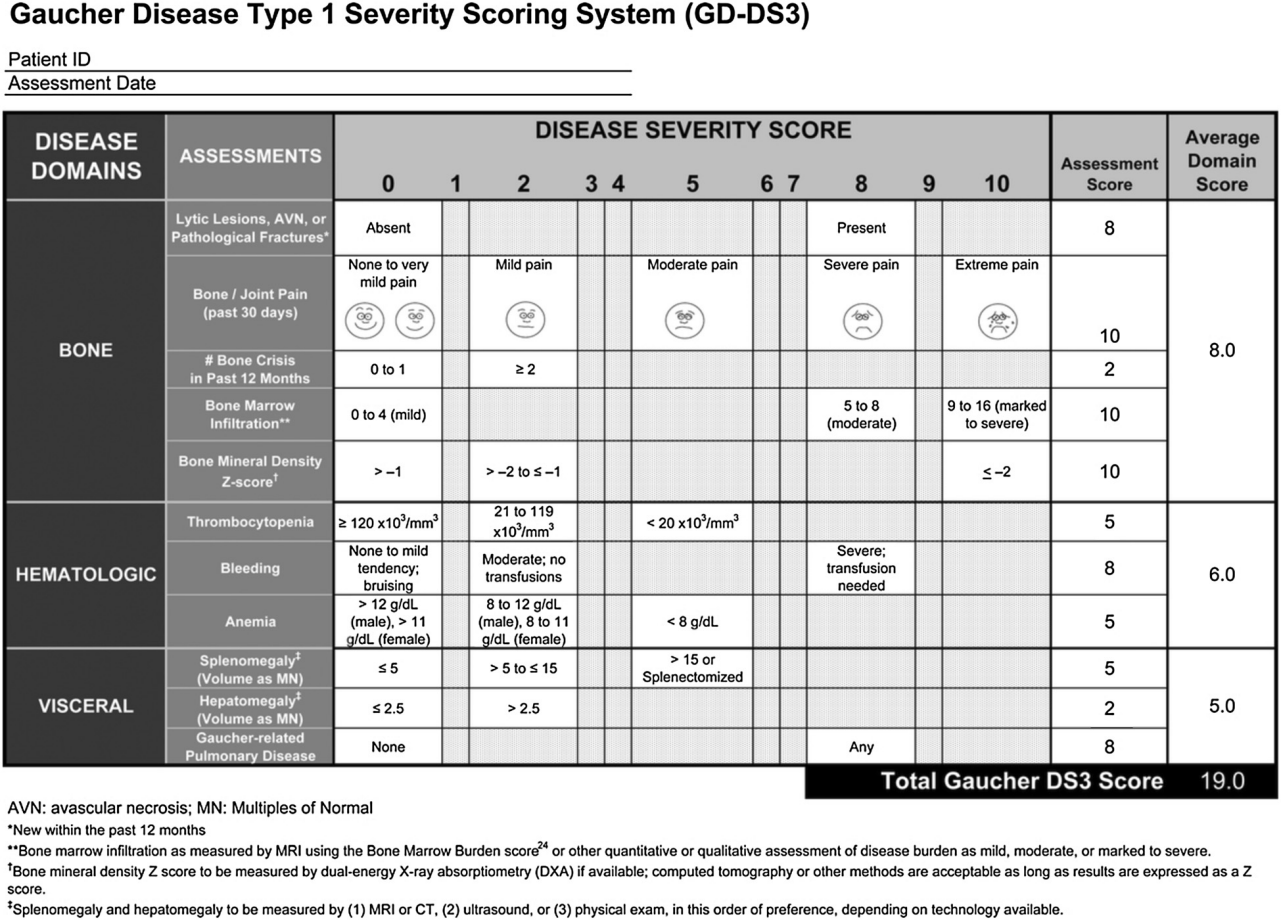
GD-DS3.

Patients were categorized based on a validated DS3 disease severity scoring system [17], performed according to instructions in Figure 1 and evaluated per instructions in Table 1. For treated patients, baseline DS3 scores were calculated just prior to or at the time of initiation of enzyme replacement therapy (ERT). For patients never treated with ERT, the DS3 score was calculated at the most recent follow up point for which complete data was available.

**Table 1:** Gaucher disease type 1 DS3 scoring

**General instructions**	
	1. Record date of assessment
	2. For each assessment, determine the GD1 DS3 score of the patient at the time of evaluation (See notes below regarding specific assessments).
	a. If current data are not available for all assessments when the DS3 score is calculated, data from previous evaluations may be used if the patient’s overall clinical status has remained stable and assessments were collected within the following period of time prior to the current date:
	• Bone imaging 12–24 months
	• Hematological 12–24 months
	• Visceral imaging 12–24 months
	b. If bone marrow infiltration and/or bone mineral density data are not available at the time of assessment or from previous evaluations, the GD1 has been optimized to be accurate and consistent without these parameters.
	c. All other assessment scores within the time frames described above are required.
DS3 score calculation	
	1. First calculate the average Disease Domain Scores by adding the assessment scores for each domain (bone, haematological, visceral) and dividing by the number of assessment scores completed. Do not include assessments that were marked “not available” (NA)
	2. The total GD1 DS3 score is the sum of the three Disease Domain Scores.
Maximum possible DS3 score	
	1. The maximum possible DS3 score is 19.
	2. In initial validation testing using 20 patient cases scored at 2 different time points, no patient received a score higher than 13 and scores above 9 correlated with an expert assessment of “severe disease”.
Interpretation of GD1 DS3 scores	
	1. 0-3 Borderline to mild disease
	2. 3–6 Moderate disease
	3. 6–9 Marked disease
	4. >9 Severe disease
Notes regarding specific assessments	
	1. Lytic lesions, AVN or pathologic fracture “present” means any new occurrence in the past 12 months.
	2. Bone marrow infiltration may be reported either semi-quantitatively (BMB score) or qualitatively (mild, moderate, marked to severe.
	3. For bleeding, an assessment of moderate (no transfusions) or severe (transfusion needed) should be based on bleeding considered by the assessor to be related to GD, whether due to low platelet count, other hemostatic disorders or vascular disease such as portal hypertension.
	4. Assessment of bone pain should be based on severity in the absence of analgesics and should consider only pain resulting from GD rather than pain attributable to other concurrent musculoskeletal diseases.

### Therapeutic goals

In 2008, an ICGG Registry benchmark analysis evaluated the attainment of six previously suggested therapeutic goals (for hemoglobin concentration, platelet count, spleen volume, liver volume, bone pain, and bone crisis) in 195 non-splenectomized patients with type I GD after 4 years of imiglucerase treatment [18]. Here, we present therapeutic outcomes relative to the same therapeutic goals for our cohort of patients treated with enzyme replacement therapy for a minimum of 3 years. Results are reported for the date of latest follow up and separately for patients with a history of pre-treatment splenectomy.

### Statistical analyses

Demographic analyses used standard descriptive statistics (frequencies and percentages). DS3 scores are summarized using means and standard deviations. ANOVA testing was performed using a 2 tailed *T* test (Excel, Microsoft Corporation).

## Results

### Demographics and genotype

93 individuals (mean age 62.1 years; range 25–91) were initially eligible. 57 (61.2%) were women. The mean (SD) age at first assessment was 49.9 years (SD 19, range: 4.2 to 83.6y). 75 patients (80.6%) reported Ashkenazi Jewish ethnicity. Five lacked genotype information. There were 4 sibling pairs. Of 84 unrelated patients with genotype data, 52 (61.9%) were homozygous for the N370S allele and 29 (34.5%) carried one N370S allele (Table 2). Among patients of Ashkenazi Jewish ethnicity, 51 of 71 with known genotypes were N370S homozygous (71.8%) while only 2 of 17 non-Jewish patients (11.8%) were homozygous for N370S.

**Table 2:** GBA genotypes and ethnicity of South Florida patients with GD1

** GBA genotype**	**Ashkenazi Jewish**	**Non-Ashkenazi Jewish: Hispanic**	**Non-Ashkenazi Jewish: Caucasian**
N370S/N370S	51*		2
N370S/V394L	5*		
N370S/84GG	4		
N370S/IVS^2+1^	2		
N370S/L444P	2	2	7*
N370S/other	5*		5
Other/other	2	1	
Unknown	4		1

*Includes 1 sibling pair.

**Figure 2:**
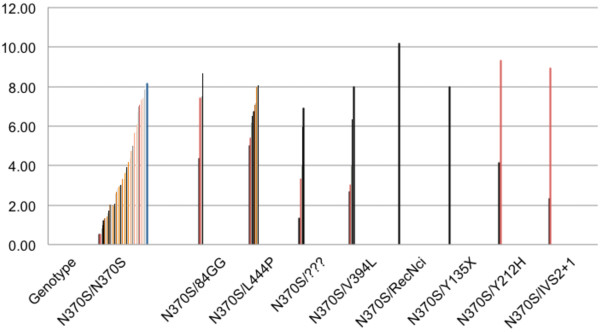
DS3 scores (Y axis) per GBA genotypes in 89 South Florida patients with GD1.

For the 88 patients with known genotypes, sufficient information to calculate baseline DS3 scores was available for 81 patients. DS3 scores in N370S homozygous patients (N = 48) were highly variable but generally in the mild to moderate range (Table 3). Because of small numbers of patients in each sub-group, the only genotype with a statistically significant difference in DS3 score from N370S/N370S was N370S/L444P (N = 10) in which severity was generally severe. Severity was also significantly higher when N370S/84GG patients (N = 4) are grouped with the N370S/L444P patients. The broad range of genotypes and disease severity in South Florida patients is shown is shown in Figure 2 which emphasizes the highly variable severity scores in N370S homozygous patients with approximately half the patients falling within the moderate to marked severity range.

**Table 3:** South Florida GD1 patients: Mean DS3 scores per genotype category

**Genotype**	**Mean DS3 score (SD)**	**95% CI**	**P value (*T *** **test)**
**N370S/N370S (N = 48)**	**3.86 (2.31)**	**3.21-4.51**	**Reference**
N370S/84 GG compound heterozygote (N = 4)	6.98 (1.85)	5.17-8.79	NS
N370S/L444P (N = 10)	6.82 (1.08)	6.15-7.49	0.018
N370S/unidentified (N = 5)	4.30 (2.20)	2.37-6.23	NS
N370S/V394L (N = 5)	4.80 (2.29)	2.79-6.81	NS
N370S/L444P or 84GG compound heterozygotes and (N = 14)	6.86 (1.26	5.60-8.12	0.021
N370S/Y212H (N = 2 sisters)	9.33; 4.17		
N370S/IVS2 + 1 (N = 2)	8.93; 2.33		
N370S/F216Y (N = 1)	1.83		
N370S/RecNCI1 (N = 1)	10.17		
N370S/Y135X (N = 1)	8.00		
R463C/K198 (N = 1)	5.08		
L444P/unidentified (N = 1)	2.50		

Statistical comparison is for the N370S/N370S genotype versus the other genotypes identified.

### Treatment status (93 patients)

Fifteen patients have not received disease-specific treatment primarily because comprehensive initial and serial assessments (NJW) indicated a clinically mild phenotype. ERT access was not problematical. One patient with symptomatic bone disease declined ERT and SRT. Until the imiglucerase shortage/interruption beginning in June 2009, virtually all patients who required treatment received imiglucerase. Two patients of unknown genotype who died prior to 1995 received alglucerase 60 U/kg every 2 weeks. They are excluded from the genotype and response analyses . At last evaluation through June 2011, 49 patients were receiving imiglucerase of whom 1 was infused weekly, 33 every two weeks, 2 every three weeks and 13 every four weeks (Table 4). Doses ranged from 20–120 U/kg but most received 30–60 U/kg. Ten patients received velaglucerase alfa mostly every 2 weeks at 60 U/kg. Four patients were treated with oral miglustat 100 mg three times daily. 13 patients had treatment interruptions– 3 for infusion reactions prior to 2009 and 10 due to the imiglucerase shortage. One has been stable since splenectomy in 1997 and the other 12 are clinically stable.

**Table 4:** Treatment Status of 91 South Florida GD1 patients as of the most recent evaluation through June 2011*

	**No. of pts**	**ERT dose (Units/kg)**			
**Treatment status**		**<30**	**30-45**	**46-60**	**>60**
Never treated	15				
Imiglucerase (total)	49				
Q1W				1	
Q2W		3	14	16	
Q3W				2	
Q4W			1	9	3
Velaglucerase alfa (total	10				
Q2W			2	6	
Q3W					1
Q4W				1	
Miglustat 100 mg po TID	4				
Treatment interrupted and not resumed (total)	13				
Severe infusion reactions	3				
Treatment shortage	10				

*Two patients with unknown genotypes who were treated solely with alglucerase prior to 1994 are excluded from this analysis.

### Treatment outcomes and attainment of therapeutic goals

Of the initial 93 patients, 61 are included in this analysis. Fifteen untreated patients, 12 patients treated for less than 3 years, and 5 patients with insufficient data were excluded. The median treatment duration at the time of analysis is 12 years (Range: 3-19y; Mid-quartile range: 6.6-16.5y). Fifteen patients (24.5%) had splenectomy prior to treatment initiation.

17 patients are deceased of whom 4 had undergone splenectomy. The mean age (SD) at death was 71.9 (15.4) years; median age at death (range): 71.5 years (28y-92y); interquartile range: 64.5y-83.8y. The genotypes of deceased patients were 13 - N370S/N370S, 2 N370S/L444P and 1 N370S/V394L. The causes of death are shown in Table 5. The mean pre-treatment DS3 score for the 13 N370S homozygous deceased patients is 5.50 (SD 1.79) compared to 3.22 (SD 2.05) for the treated N370S homozygous patients who remain alive (p = 0.002). The mean age (SD) at which ERT was initiated was 66.0 (SD 10.6) years for the deceased N370S homozygous patients and 52.1 (SD 14.4) years for the still surviving N370S homozygous patients (p = 0.006).

**Table 5:** Causes of death in 17 deceased South Florida treated GD1 patients

** Cause of death**	**Total number of pts**	**Number of pts with splenectomy**
Parkinsonism*	3	2
Chronic kidney disease	3	
Acute myeloid leukemia and/or myelodysplasia	3	1
Dementia without Parkinsonism	2	
Non-Hodgkin’s lymphoma	1	1
Lung cancer, metastatic	1	
Cerebrovascular accident	1	
Congestive heart failure	1	
Bronchiolitis obliterans and auto-immune hemolytic anemia	1	
Air embolism associated with illicit IV drug use	1	

*Age at Parkinson disease diagnosis: 45, 54, and 63 years respectively.

As shown in Table 6, 26 concurrent malignancies developed in 24 of 93 patients (25.8%). Two patients had 2 cancers (cutaneous melanoma and localized breast CA; cutaneous squamous cell CA and cervical CA in situ). Four patients have a significant monoclonal gammopathy but none has yet developed overt multiple myeloma. Cancers were diagnosed at a median age of 67 years (47–83 y). Of the 24 patients with concurrent malignancies, 5 have never been treated definitively for GD1 (Table 4).

**Table 6:** Concurrent malignancies in 93 South Florida patients with GD1

** Malignancy**	**No of patients**	** Ages at CA Dx (y)**	**Mean age at CA Dx (y)**
Monoclonal gammopathy	4	54, 68, 70^#^, 82^#^	68.5
Cutaneous Squamous CA	5	59, 66, 66, 77, 80	69.6
Myelodysplasia, AML	3	51*, 63*, 72*	62.0
Non-Hodgkin’s lymphoma	3	59, 74*, 83	72.0
Breast carcinoma	3	47^#^, 72^#,^ 75	64.7
Cutaneous Melanoma	2	57, 71	64.0
Prostate carcinoma	2	65, 75	70.0
Bladder carcinoma	1	80	80.0
Lung cancer	1	62*	62.0
Fallopian tube carcinoma	1	49^§#^	49.0
Cervical carcinoma in situ	1	53	53.0

*Cause of death.

^§^BRCA2 mutation.

^#^Never treated with ERT or SSIT.

After a median 12 treatment years, South Florida patients matched or exceeded the ICCG 4 year therapeutic goal achievement for platelet count (85.4% vs. 79.6% success), spleen volume (93.3% vs. 78.0% success), liver volume (93.4% vs. 90.6% success), and bone crises (100% vs. 99% success). Nevertheless, fewer patients with intact spleens had sustained achievement of all 6 therapeutic goals (30.4% versus 41.4%) and only 40% of the splenectomy patients sustained achievement of 5/5 possible goals (Table 7). Of 6 South Florida patients who “failed” therapeutic goal criteria for hemoglobin concentration, 5 had concurrent hematological diagnoses: auto-immune hemolytic anemia [1], acute myelocytic leukemia [1], myelodysplasia [1], chronic kidney disease [2].

**Table 7:** **Attainment of therapeutic goals: South Florida patients with GD1 and the ICCG benchmark cohort**[18]

** Therapeutic goal**	**South Florida GD1 patients (Median treatment:12 years minimum treatment: 3 years)**	**ICGG cohort (N-195) (evaluated after 4 years of ERT)**
Bone pain (N = 61)	45.3%^#^	70.2%^#^
Hemoglobin (N = 61)	90.6%*	91.6%
^§^Platelets (N = 46)	85.4%	79.6%
^§^Spleen volume (N = 46)	93.3%	78.0%
Liver volume (N = 61)	93.4%	90.6%
Bone crises (N = 61)	100%	99.9%
^§^Attained 6/6 goals (N = 46)	30.4%	41.4%
^§§^Attained 5/5 goals (N = 15)	40.0%	Not applicable

^§^Intact spleen patients only.

^§§^Splenectomy patients.

^#^63% of the ICGG patient cohort had **no** reported bone pain prior to initiation of treatment. 73.4% of the South Florida patients reported bone pain prior to initiation of treatment; bone pain was scaled as moderate or worse in 59.6%.

*In 5/6 patients, Hb was depressed because of concurrent illnesses at evaluation point (auto-immune hemolytic anemia, acute myeloid leukemia, myelodysplasia, chronic kidney disease [2]).

54.7% of the intact spleen patients (and 53.3% of the splenectomy patients, for whom there is no comparison group) continued to have bone pain vs. 29.8% in ICCG. On average, there was no age difference between patients with residual bone pain and those free of pain. Of note, only 37% of the ICGG patient cohort had bone pain prior to initiation of treatment compared to 73.4% of the South Florida patients (moderate or severe pain in 59.6%). Of 28 patients with baseline bone pain scaled as moderate or worse, 20 (71.4%) failed to realize the therapeutic goal for bone pain at their most recent evaluation. Of 35 patients who initially reported either no bone pain or pain no worse than mild, 15 (42.8%) had bone pain at their most recent evaluation. Age at inception of ERT was not significantly associated either with severity of pre-treatment bone pain or with therapeutic goal “failure” due to persistent bone pain. However, of the 61 patients included in the therapeutic goal analysis, only 9 patients were younger than 30 years of age when ERT was begun and only 4 were younger than 18 years. Regardless of age at inception of treatment, severity of pre-treatment bone pain was a key predictor for persistent bone pain. Age at commencement of ERT also did not appear to be a significant determinant of failure to achieve therapeutic goals other than bone pain. Among our patients, poor compliance with treatment schedules was not a contributory factor to poor therapeutic responses.

The initial total DS3 bone domain score, although including heavily weighted information about avascular necrosis, lytic lesions, fractures, bone marrow infiltration and bone density, was less likely to predict pain outcomes than the bone pain score itself. There also was no significant association between the baseline DS3 bone domain score and composite attainment of therapeutic goals in either patients with intact spleens or in those with splenectomy (Table 8). Likewise, neither the total pre-treatment DS3 score nor the GBA genotype was predictive of outcome in terms of achievement of the 6 therapeutic goals that were examined.

**Table 8:** Lack of association between the baseline DS3 bone domain score and composite attainment of therapeutic goals

**Therapeutic goals attained**	**Number of patients**	**Mean DS3 score (SD)**	**95% Confidence interval**	**ANOVA P value**
Intact spleen pts (N = 45)				0.508
6 of 6	14	2.70 (1.60)	1.86--3.54	
5 of 6	21	3.01 (1.71)	2.28--3.74	
4 of 6	10	2.23 (1.97)	1.01--4.20	
Splenectomy pts (N = 15)				0.282
5 of 5	6	4.26 (2.33)	2.40--6.12	
4 of 5	5	5.82 (1.03)	4.92--6.72	
3 of 5	3	6.08 (1.28)	4.63--7.53	

## Discussion

To our best estimate, the 93 patients included in this report constitute at least 75% of known GD1 patients living in South Florida during a 22 year period. During that time, a 1990 population of 3.5 million in which 57% were non-Hispanic White has grown to 5.5 million with only 41% being non-Hispanic White [19]. There are approximately 2.3 million Hispanic or Latino South Floridians of any race and 1.1 million of Black-American ethnicity. 37% of the region’s population is foreign-born and 32% were born elsewhere in the United States. Nevertheless, our patients with GD1 continue to be genotypically and phenotypically different from patient populations that have been reported from various European and Latin American countries and much closer in characteristics to other patients with GD1 living primarily in the northeastern United States, California and in Israel (Table 9).

**Table 9:** GD1 Genotypes in different world populations

**Country or Region**	**N370S/N370S (%)**	**N370S/L444P (%)**	**N370S/other (%)**	**Total N370S (%)**
Spain and Portugal (N = 370)	16.8	31.6	43.2	91.6
France* (N = 203)	19.2	20.2	60.6	?
Netherlands (N = 40 unrelated)	2.5	40.0	45.0	87.5
Italy (N = 106 unrelated)	12.3	25.4	49.1	86.8
UK and Ireland (N = 30)	30.3	3.3	40.0	73.6
Turkey (N = 32)	31.2	18.8	25.0	75.0
Latin America (N = 431)	11.7	29.3	52.0	93.0
World ICGG Registry 1998 (N = 680)	24.0	18.1	46.7	88.8
Jewish: USA and Israel (N = 545 unrelated)^§^	45.0	8.8	34.1	87.9
AZ CT NJ NY, USA (N = 403)	53.6	13.4	33.0	100.0
South Florida, USA (N = 84 unrelated)	61.9	11.9	22.6	96.4

*Restricted to GD1 patients with at least one N370S allele.

^§^From Boot RG et al. Human Mutation. 10:348:1997.

Compared to the GD1 patients from the Northeast United States described by Taddei et al. [11], somewhat fewer South Florida patients were Ashkenazi Jews, were N370S homozygous, and had a history of splenectomy. Our patients were significantly older when initially assessed (mean age 49.9 years (SD 19.0) versus 39.2 years (SD 18.8), p < 0.0001). The difference in age and increased years at risk may explain why the occurrence of malignancy was greater in our patient group (24/93 (25.8%) versus 46/367 (12.0%)). Among our patients with cancers, hematological malignancies were the most prevalent. However, despite strong evidence for a substantial increased risk for this malignancy in older patients with GD1 [20-23], none of our patients have yet developed myeloma although four have monoclonal gammopathy of uncertain significance. Our experience does not suggest that patients with GD1 have a greater risk for developing the most common solid tumors (lung, breast, prostate, colorectal, pancreatic) than unaffected individuals in the general population.

With the exception of N370S/N370S, the number of our patients in each GBA1 genotype sub-set is small. Nevertheless, the DS3 scores are consistent with the consensus perception of “severity” of GD1 genotypes and our results complement the validity testing of the DS3 instrument [17]. In most western non-Jewish populations, 20-30% of patients with GD1 are N370S/L444P and moderately to severely affected prior to starting treatment. The mean DS3 score for our N370S/L444P patients (6.82) is consistent with this experience. No N370S/L444P patient in our study had a pre-treatment DS3 score in the mild range. There was more DS3 score heterogeneity among other N370S heteroallele genotypes including N370S/V394L (generally associated with mild phenotypes) [24] and N370S/IVS^2+1^ (generally associated with clinically more severe disease [25].

As expected, there was substantial variability in the severity scores among N370S homozygous patients [26]. Although the lowest DS3 scores (<2.00) were confined to the N370S/N370S patients, half of the N370S homozygous patients had DS3 scores that were in the moderate range or higher. Skeletal disease, as measured with the Hermann score and Zimran Severity Score Index (SSI) is reported to worsen with age in untreated, non-splenectomized homozygous GD1 patients [11]. However, in similar patients, we did not find a significant correlation between patient age at first assessment and either bone domain DS3 score (ρ = 0.21) or total DS3 score (ρ = 0.25).

As regards treatment response, pending completion of a larger, multicenter study designed to examine serial changes in DS3 scores in both treated and untreated patients (Clinicaltrials.gov; NCT01136304), we elected to evaluate our patients in terms of achievement of therapeutic goals for hemoglobin concentration, platelet count, spleen and liver enlargement, bone pain and bone crises. These short term goals are not necessarily disease specific over an extended period of observation and they are an incomplete representation of all the elements that are integral to capturing the essence of clinical response to treatment for life long chronic illnesses such as GD1. Nevertheless, regulatory approvals of new therapy for GD1 and most reports of 4–10 year treatment outcomes have relied heavily, and sometimes exclusively on these six parameters [18,27], and sustained cumulative maintenance of these specific therapeutic goals has been proposed as a benchmark for therapeutic efficacy and as a basis for treatment comparisons [28].

Although assessment of achievement of multiple therapeutic goals is very useful for individualized case management, our results demonstrate the limitations and pitfalls of such applications to studies of patient populations that are not matched in terms of pre-treatment characteristics and potentially confounding concurrent or emerging medical events. Although the percentages of our patients completely at therapeutic goal after a median 12 years of GD1 treatment was less than that reported by ICCG after 4 years of treatment, the clinical circumstances suggested that many “failures” were probably not attributable to “breakthrough” manifestations of GD1. In fact, with the longer duration of treatment, as predicted by the 10 year imiglucerase experience [27], improvement in thrombocytopenia and regression of hepatosplenomegaly exceeded that observed in the ICGG study. Recurrent anemia was associated with onset of concurrent illnesses including renal insufficiency, GI bleeding, or hematologic malignancy and risk of death was clearly associated with aging and cumulative co-morbidities. Persistent bone pain, the greatest cause for failure to achieve all therapeutic goals, is difficult to interpret objectively. Although chronic pre-treatment bone pain was often persistent, we found that there was little correlation with the presence of objective evidence of bone damage such as infarction, osteonecrosis or prior fractures, with severity of bone marrow infiltration and osteopenia, or with overall DS3 severity score. As patients age, it becomes more difficult to distinguish between Gaucher bone pain and that attributable to other musculoskeletal diagnoses. Unresponsive pain in young individuals is sometimes associated with drug-seeking behaviors not necessarily related to severity of GD1 based on objective measurements. In a scientific study, can an investigator legitimately dismiss positive patient pain reports of uncertain relevance, without introducing the possibility of bias? If not, are the resultant aggregate “report cards” an accurate measure of outcome? The contextual relevance of bone pain reports would be enhanced by accompanying assessments of duration and constancy of pain, effects on activities of daily living including school and work performance and fluctuations in the use of adjuvant analgesic medications.

The clinical outcomes research field is increasingly emphasizing the centrality of patient-reported input and value-based medicine (the ability of an intervention to produce a clinical benefit in actual practice) as a necessary accompaniment to classical efficacy and safety studies with physician-conceived endpoints (evidence-based medicine) [29,30]. Rare, chronic, phenotypically heterogeneous diseases such as GD1 for which treatment, when indicated, is generally lifelong and very expensive, are prime targets for value-based research studies that are applicable not only to individual patient care choices but also to justification of societal health care priorities. Our experiences with the medical complexity of a “simple Mendelian hereditary disease” population [31], with the confounding effects of age-acquired co-morbidities on even precisely defined and measured end points such as hemoglobin concentration and with the difficulties in contextual interpretation of patient-reported symptoms such as bone pain highlight the need for expert guidance when implementing patient-centered outcomes research [32]. Although such studies strive to maximize patient creativity and freedom of expression, they nonetheless require careful design and annotation of patient characteristics, clear objectives, defined nomenclature, and formulation of focused and unambiguous questions that are meaningful and important to the participants but whose answers will be internally consistent and amenable to rigorous analysis.

## Competing interests

Neal J Weinreb receives honoraria and expense reimbursement for serving on a Board of Advisors of the ICGG Gaucher Registry; travel reimbursements and/or honoraria and/or research support from Genzyme-a Sanofi Company, Shire, Pfizer Corporation, and Actelion Corporation. Olaf A Bodamer is member of the Speaker Bureau for Shire and Genzyme-Sanofi, receives research support from Shire and is on the US Advisory Board for Gaucher Disease for Shire.

## Authors’ contributions

MO participated in data analysis and helped to draft and to revise the manuscript. DB participated in the study design and data analysis and helped revise the manuscript. OAF helped to review and revise the manuscript. NJW conceived of the study, participated in the study design, data analysis and statistical analysis, and helped to draft and to revise the manuscript. All the authors read and approved the final manuscript.
